# Sucrose synthases are not involved in starch synthesis in *Arabidopsis* leaves

**DOI:** 10.1038/s41477-022-01140-y

**Published:** 2022-04-28

**Authors:** Maximilian M. F. F. Fünfgeld, Wei Wang, Hirofumi Ishihara, Stéphanie Arrivault, Regina Feil, Alison M. Smith, Mark Stitt, John E. Lunn, Totte Niittylä

**Affiliations:** 1 Max Planck Institute of Molecular Plant Physiology, Potsdam-Golm, Germany; 2 Department of Forest Genetics and Plant Physiology, Swedish University of Agricultural Sciences, Umeå Plant Science Centre, Umeå, Sweden; 3 John Innes Centre, Norwich Research Park, Norwich, UK; 4 Present Address: Luxembourg Institute of Health, Strassen, Luxembourg; 5 Present Address: University of Helsinki, Helsinki, Finland

**Keywords:** Plant physiology, Plant molecular biology

## Abstract

Many plants accumulate transitory starch reserves in their leaves during the day to buffer their carbohydrate supply against fluctuating light conditions, and to provide carbon and energy for survival at night. It is universally accepted that transitory starch is synthesized from ADP-glucose (ADPG) in the chloroplasts. However, the consensus that ADPG is made in the chloroplasts by ADPG pyrophosphorylase has been challenged by a controversial proposal that ADPG is made primarily in the cytosol, probably by sucrose synthase (SUS), and then imported into the chloroplasts. To resolve this long-standing controversy, we critically re-examined the experimental evidence that appears to conflict with the consensus pathway. We show that when precautions are taken to avoid artefactual changes during leaf sampling, *Arabidopsis thaliana* mutants that lack SUS activity in mesophyll cells (quadruple *sus1234*) or have no SUS activity (sextuple *sus123456*) have wild-type levels of ADPG and starch, while ADPG is 20 times lower in the *pgm* and *adg1* mutants that are blocked in the consensus chloroplastic pathway of starch synthesis. We conclude that the ADPG needed for starch synthesis in leaves is synthesized primarily by ADPG pyrophosphorylase in the chloroplasts.

## Abstract (web summary)

Mutant analysis shows that sucrose synthase makes no substantial contribution to transitory starch synthesis in *Arabidopsis* leaves, resolving a 20-year-old controversy about one of the most important pathways of photosynthetic metabolism.

## Main

Many plants accumulate starch reserves in their leaves during the day, which are remobilized at night to provide carbon and energy for survival at night^1,2^. It is universally accepted that starch is synthesized from ADP-glucose (ADPG) and that in leaves starch is synthesized in the chloroplasts, primarily in mesophyll cells. However, there is disagreement about the subcellular compartmentation and pathway of ADPG synthesis. Studies on isolated chloroplasts^3,4^ and starch-deficient mutants of *Arabidopsis thaliana*^5–11^ led to a consensus view that ADPG is produced in the chloroplasts by ADPG pyrophosphorylase (AGPase), with its glucose 1-phosphate (Glc1P) substrate being derived from fructose 6-phosphate (Fru6P) that is withdrawn from the Calvin-Benson cycle in the light, and converted sequentially to glucose 6-phosphate (Glc6P) and then Glc1P by the plastidial phosphoglucose isomerase (PGI) and phosphoglucomutase (PGM), respectively. This pathway is consistent with the near absence of starch in source leaves of *pgm* and *adg1* null mutants that lack plastidial PGM or AGPase, respectively^5–7^. ADPG and starch synthesis are thought to be restricted to the chloroplasts in leaves of all species and to the amyloplasts in non-photosynthetic tissues (for example, *Arabidopsis* seeds), except for the endosperm of cereals and other Poaceae^12,13^. Here the majority of ADPG is synthesized by a cytosolic AGPase and imported into the amyloplasts via BRITTLE1 (BT1)-type ADPG transporters, probably in exchange for AMP^14–19^. In barley (*Hordeum vulgare*) and rice (*Oryza sativa*), alternative splicing of the *AGPS* small subunit gene in different tissues leads to expression of a chloroplast-targeted AGPS polypeptide in leaves, while the AGPS polypeptide expressed in the endosperm has no plastidial transit peptide and is located in the cytosol^20,21^. In maize (*Zea mays*) endosperm, the cytosolic AGPase derives from gene duplication and neo-functionalization during tetraploidization^22^.

**Fig. 1:**
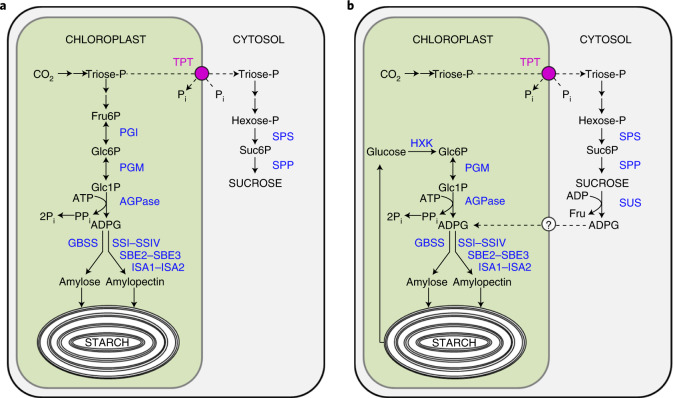
Starch synthesis in *Arabidopsis* leaves. **a**, The classical pathway of starch synthesis (modified from a figure in ref. ^68^) in which the substrate for starch synthesis, ADP-glucose (ADPG), is synthesized in the chloroplasts by ADP-glucose pyrophosphorylase (AGPase). **b**, The alternative pathway proposed by Baroja-Fernández et al.^23^ in which ADPG is primarily produced in the cytosol via sucrose synthase (SUS) and imported into the chloroplasts via a so far unknown transporter for starch synthesis. The model proposes that starch is turned over hydrolytically during the day producing glucose, which is phosphorylated by hexokinase (HXK) and converted back into ADPG via plastidial phosphoglucomutase (pPGM) and ADP-glucose pyrophosphorylase for resynthesis of starch. Fru6P, fructose 6-phosphate; GBSS, granule-bound starch synthase; Glc1P, glucose 1-phosphate; Glc6P, glucose 6-phosphate; ISA1, isoamylase 1; ISA2, isoamylase 2; PGI, plastidial phosphoglucose isomerase; PGM, plastidial phosphoglucomutase; SBE2-SBE3, starch branching enzymes 2–3; SPP, sucrose-phosphate phosphatase; SPS, sucrose-phosphate synthase; SSI-SSIV, soluble starch synthases 1–4; Suc6P, sucrose 6´-phosphate; TPT, triose-phosphate translocator.

This consensus has been challenged by a proposal that ADPG is synthesized primarily by sucrose synthase (SUS) in the cytosol, and then imported into the chloroplasts for starch synthesis (Fig. 1b)^23–29^. In addition to uridine diphosphate (UDP), the preferred substrate, SUS can use ADP as an alternative substrate, producing ADPG instead of UDPG as a product of sucrose cleavage^30^. The proposed SUS-mediated pathway of ADPG synthesis was based on two lines of evidence. First, if the consensus pathway operates, levels of ADPG and starch would be expected to be much lower in leaves lacking PGM or AGPase (the *pgm* and *adg1* (*aps1*) mutants, respectively) than in wild-type (WT) leaves. This was claimed not to be the case: ADPG levels in *pgm* and *adg1* mutants were reported to be similar to those in WT plants^28,29^. Second, in an attempt to discover the effects of reductions in ADPG levels on starch synthesis in either the chloroplast or the cytosol on starch synthesis, Baroja-Fernández et al.^24^ expressed an adenosine diphosphate sugar pyrophosphatase (ASPP) from *Escherichia coli* in one or the other of these compartments in WT plants. This enzyme cleaved ADPG into Glc1P and AMP, but could also hydrolyze other ADP-sugars, with ADP-ribose being the preferred substrate^31^. Expression of ASPP in chloroplasts had little effect on ADPG levels, but reduced starch accumulation by 50%. Expression in the cytosol reduced ADPG levels by 30% and starch levels by 20%^24^. From these observations, it was argued that a pool of ADPG located in the cytosol is essential for normal rates of starch synthesis. As AGPase is strictly chloroplastic in *Arabidopsis* leaves^32,33^, Baroja-Fernández and colleagues proposed that SUS synthesizes ADPG in the cytosol, using ADP as the glucosyl acceptor in the sucrose-cleavage reaction, followed by transfer of ADPG into the chloroplast via a hypothetical transporter in the chloroplast envelope^23^. However, it remains possible that the reduction in starch levels in plants with cytosolic ASPP is a pleiotropic effect, brought about by ASPP-mediated changes in ADP-sugars other than ADPG.

The proposed SUS pathway for ADPG synthesis has been critically discussed by Okita^34^ and Neuhaus et al.^35^. One of their major criticisms was that the SUS pathway, as originally proposed, did not explain the starch-deficient phenotypes of the *pgm* and *adg1* mutants. In response to this criticism, it was proposed that a futile cycle of starch synthesis and degradation operates inside the chloroplast during the day, in parallel with synthesis and import of cytosolic ADPG. In the proposed futile cycle, starch is degraded hydrolytically to glucose, which is then converted via plastidial hexokinase, PGM and plastidial AGPase to ADPG for resynthesis of starch^24,27^. According to this scheme, loss of either plastidial PGM or AGPase would prevent recycling of the products of starch degradation and thus prevent starch accumulation. Such futile cycling would need to operate under all conditions and throughout the photoperiod to explain the starch-deficient phenotypes of the *pgm* and *adg1* mutants.

The proposal that futile cycling of starch occurs continuously in the light is called into question by several observations. First, recent ^13^C-labelling studies have shown that there is some turnover of starch in *Arabidopsis* leaves in the light, but this is limited to certain times of day and prevalent only in long-day conditions^36^. Second, Streb et al.^37^ introgressed the *pgm* mutation into null mutants for key steps in the major pathway of starch degradation in *Arabidopsis* leaves^2,38^: glucan, water dikinase (GWD; encoded by *STARCH EXCESS 1*), phosphoglucan phosphatase (*SEX4*), isoamylase3 (*ISA3*) debranching enzyme and the maltose transporter (*MEX1*). All of the resulting double mutants were as starch-deficient as *pgm*, consistent with a requirement for plastidial PGM in the primary pathway of starch synthesis. Third, even if the proposed futile cycling of starch and glucose salvage pathways were operating, the SUS pathway does not readily explain the starch-deficient phenotype of *Arabidopsis* plastidial *pgi* mutants^9^. Proponents of the SUS pathway argue that plastidial *pgi* mutants are starch-deficient because of a defect in the synthesis of isoprenoid-derived phytohormones (for example, gibberellins) that perturbs their growth and seed production, rather than a simple metabolic block in the flow of carbon from Fru6P, an intermediate of the Calvin-Benson cycle, to ADPG^39^. Fourth, the SUS pathway also does not explain why *triose-phosphate translocator* (*tpt*) mutants, which are unable to export triose-phosphates for sucrose biosynthesis, accumulate more starch than WT plants in the light^40,41^. Likewise, mutants defective in the pathway of sucrose synthesis usually have elevated starch^42–45^. If ADPG were synthesized primarily from sucrose via SUS, all of these mutants would be expected to have less starch than WT. Furthermore, *f2kp* mutants that lack fructose-2,6-bisphosphate have enhanced rates of sucrose synthesis but less starch than WT plants^46^, not more as would be expected if ADPG and starch were derived from sucrose. Fifth, there is no experimental evidence that *Arabidopsis* leaf chloroplasts have the capacity to import ADPG from the cytosol. On the contrary, eudicot forms of the plastidial BRITTLE1 adenylate transporter, including the *Arabidopsis* orthologue AtBT1, have been shown to transport AMP, ADP and ATP but not ADPG, and *Arabidopsis* *bt1* mutants have near-WT levels of starch^47^. These observations argue against import of ADPG via AtBT1 from the cytosol making a meaningful contribution to starch synthesis in *Arabidopsis* leaves.

A direct indication that SUS is not required for starch synthesis in mesophyll cells came from a study of a quadruple *sus1 sus2 sus3 sus4* (*sus1234*) mutant. This lacks all four of the SUS isoforms that are expressed in mesophyll cells and has little or no soluble SUS activity in the leaves, yet has WT levels of starch^48,49^. The main proponents of the SUS pathway confirmed the WT starch levels in the *sus1234* mutant, but argued that the result was inconclusive because, unlike Barratt et al.^48^, they observed high soluble SUS activity in the *sus1234* mutant. They suggested that SUS activity had been underestimated by Barratt et al.^48^ and that *Arabidopsis* might have other, cryptic forms of SUS^25,50^.

Barratt et al.^48^ also questioned whether WT *Arabidopsis* leaves have sufficient SUS activity to account for their rates of starch synthesis. This was based on a previous report that SUS activity in WT leaves (0.023 µmol min^−1^ g^−1^FW)^51^ was lower than the rates of starch synthesis typically observed under standard laboratory conditions (up to 0.1 µmol [Glc] min^−1^ g^−1^ FW). Bieniewska et al.^51^ measured SUS activity at 25 °C in the direction of sucrose synthesis by the production of [^14^C]sucrose from UDP-[^14^C]glucose and fructose. Baroja-Fernández et al.^25^ contended that SUS activity had been underestimated due to instability of the UDP-glucose substrate under the assay conditions. They reported an activity of 0.185 µmol min^−1^ g^−1^ FW in WT leaves, measured at 37 °C in the sucrose cleavage direction using a high-performance liquid chromatography (HPLC)-based assay for UDP-glucose and ADPG, which would be sufficient to mediate observed rates of starch synthesis. The basis for these claims was in turn questioned by studies showing that UDP-glucose is more stable at moderately alkaline pH than claimed by Baroja- Fernández et al.^25,52^. These conflicting reports leave open the question of whether there is sufficient SUS activity to sustain observed rates of starch synthesis via the proposed cytosolic pathway of ADPG synthesis.

In addition to the genetic evidence favouring the consensus pathway, there are biochemical data that conflict with the proposed SUS pathway. Non-aqueous fractionation of *Arabidopsis* rosettes showed that ADPG co-localized with the chloroplastic markers, with 100% of the total ADPG being assigned to the chloroplastic fraction in a two-compartment model (chloroplast vs cytosol)^53^. When WT *Arabidopsis* rosettes were labelled with ^13^CO_2_ under steady-state photosynthetic conditions, the pool of ADPG was labelled more rapidly than sucrose, and had kinetics similar to those of Calvin-Benson cycle intermediates^53^. These labelling patterns are consistent with direct synthesis of ADPG from intermediates of the Calvin-Benson cycle in the chloroplasts, rather than via sucrose in the cytosol.

Transitory starch is a major end product of photosynthesis, therefore uncertainty about its synthesis is a major obstacle to understanding the regulation of photosynthetic carbon metabolism and how the fixed carbon is allocated between storage and export for growth. To resolve the long-standing controversy surrounding the pathway of starch synthesis in leaves, we critically re-examine the main lines of evidence that ADPG is synthesized by SUS in the cytosol for transitory starch synthesis in the chloroplasts. Furthermore, we generated *Arabidopsis* mutants that completely lack SUS activity and report that these sextuple (*sus1 sus2 sus3 sus4 sus5 sus6*) mutants have near wild-type levels of ADPG and starch, and only mild growth phenotypes under standard laboratory growth conditions.

## Results

### Optimization of ADPG measurement in *Arabidopsis* leaves

HPLC with UV detection at 260 nm (HPLC-UV) or pulsed amperometric detection (HPLC-PAD) has been widely used to assay ADPG, including all but the most recent study^29^ claiming support for the SUS pathway. However, plant extracts contain many other types of nucleotide that absorb in the UV range^54^, and it is difficult to achieve baseline separation of ADPG from other UV-absorbing compounds, potentially leading to overestimation of ADPG. Likewise, HPLC-PAD, as used in Baroja-Fernández et al.^25^, cannot distinguish ADPG from any co-eluting molecules, so is also prone to overestimation of ADPG. HPLC coupled to tandem mass spectrometry (LC-MS/MS) offers much greater specificity and sensitivity than HPLC-UV or HPLC-PAD^55^, and has become the method of choice for assaying ADPG.

**Fig. 2:**
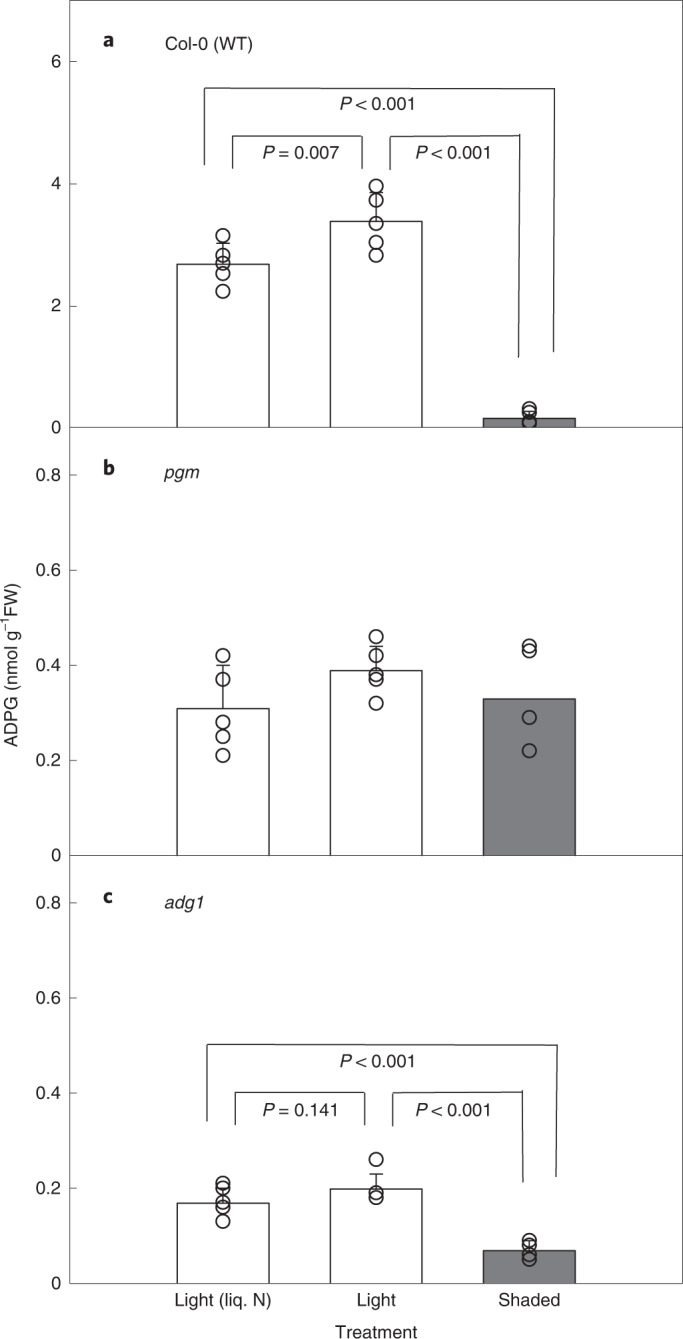
Optimization of *Arabidopsis* leaf harvesting for measurement of ADPG. **a**–**c**, Rosettes were harvested from vegetative wild-type Col-0 plants (**a**) and from starch-deficient *pgm* (**b**) and *adg1* (**c**) mutants in the light (white bars) by pouring liquid nitrogen (liq. N) directly onto the rosette or by cutting the hypocotyl from below and transferring the cut rosette into liquid nitrogen, taking care to avoid all shading. In parallel, other plants were deliberately shaded (grey bars) for 2–3 s before cutting and snap-freezing the rosettes. ADPG was measured by LC–MS/MS. Bars show mean ± s.d. of 5 independently pooled batches of 5 rosettes (*n* = 5 biological replicates). *P* values are shown for pairwise comparisons from a one-way analysis of variance (ANOVA) (Holm-Sidak post-hoc test). There were no significant differences between the samples in **b** (*P* = 0.285).

The levels of ADPG in *Arabidopsis* leaves are generally low (<5 nmol g^−1^ FW), with an estimated turnover time of <1 s in illuminated leaves^56^, suggesting that leaf sampling in the light must be done with great care to avoid artefactual decreases in ADPG content below the steady-state level before the tissue is quenched. To assess the sensitivity of ADPG to unintentional shading during leaf sampling, rosettes were harvested from WT, *pgm* and *adg1* *Arabidopsis* plants in the light either by pouring liquid nitrogen directly onto the plants, or by cutting the hypocotyl from below and then quickly transferring the cut rosette into liquid nitrogen while in the growth chamber, taking care not to shade the leaves at any time during the procedure. In parallel, rosettes were harvested from plants that had been deliberately shaded by transfer from 160 µE m^−2^ s^−1^ to <10 µE m^−2^ s^−1^ for 2–3 s. This brief shading treatment led to a large (94%) fall in rosette ADPG levels (measured by LC–MS/MS) compared with rosettes that had been harvested in the light without shading (Fig. 2a). The *pgm* mutant contained much lower levels of ADPG than WT plants, but there was no significant effect of shading (Fig. 2b), while the *adg1* mutant had even lower levels than *pgm* that were decreased even further by shading (Fig. 2c). These results demonstrate the sensitivity of ADPG in WT plants to artefactual changes due to shading during leaf sampling, hence the need for extremely rapid quenching under full illumination. Pouring liquid nitrogen directly onto the plants is inconvenient and potentially hazardous, so for subsequent experiments we cut the hypocotyl from below and immediately transferred the cut rosette into liquid nitrogen within the growth chamber.

### Comparison of ADPG levels in wild-type and mutant *Arabidopsis* plants

**Fig. 3:**
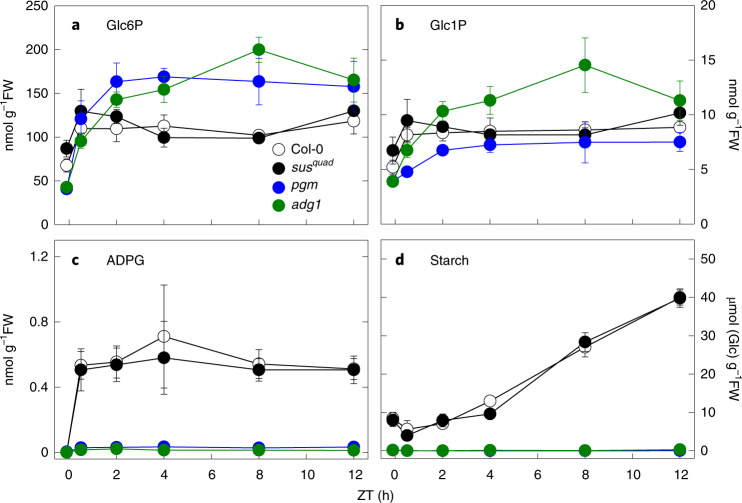
Metabolite levels in wild-type and mutant *Arabidopsis* plants. **a**–**d**, Wild-type Col-0, the *sus1234* mutant (*sus*^*quad*^) and two starch-deficient mutants (*pgm* and *adg1*) were grown in long-day conditions (16 h photoperiod). At 25 d after germination, rosettes were harvested just before dawn (ZT0.2) and at intervals from ZT0.5 to ZT12 for measurement of: (**a**) glucose 6-phosphate (Glc6P), (**b**) glucose 1-phosphate (Glc1P), (**c**) ADP-glucose (ADPG) and (**d**) starch. Data are mean ± s.d. of 4 independently pooled batches of 5 rosettes (*n* = 4 biological replicates). *P* values for all genotype × genotype comparisons are shown in Supplementary Table 2.

WT, *pgm*, *adg1* and *sus1234* plants were grown in long-day (16 h photoperiod) conditions, and rosettes were harvested at the end of the night and at intervals during the day for metabolite analysis. There were no significant differences in Glc6P and Glc1P between WT and *sus1234* plants (Fig. 3a,b and Supplementary Table 1). In the *pgm* and *adg1* mutants, Glc6P was lower than in WT in the dark, but increased to a greater extent upon illumination and was significantly higher than in WT from about Zeitgeber time (ZT) 4 (that is, 4 h after the start of the light period) onwards (Fig. 3a). Glc1P was lower in the *pgm* mutant than in WT, but higher in the *adg1* mutant than in WT (Fig. 3b). ADPG levels were extremely low (<0.005 nmol g^−1^ FW) in all genotypes in the dark (Fig. 3c). Upon illumination, ADPG increased rapidly in WT and *sus1234* plants, reaching a similar level (0.51–0.71 nmol g^−1^ FW) in the two genotypes and remaining fairly constant throughout the day with no significant differences between WT and *sus1234* plants (Fig. 3c). In contrast, ADPG rose only slightly upon illumination of *pgm* and *adg1*, reaching levels of 0.03 nmol g^−1^ FW and 0.01–0.02 nmol g^−1^ FW, respectively (Fig. 3c). The *sus1234* plants accumulated the same amounts of starch as WT plants, whereas at ZT12 the starch contents of the *pgm* and *adg1* mutants were <1% of WT (Fig. 3d). The lag in net starch accumulation in WT and *sus1234* at the beginning of the day is most likely due to starch degradation occurring in parallel with synthesis as previously observed for plants growing in long-day (16 h photoperiod) conditions^36^.

WT plants were labelled with ^13^CO_2_ under ambient CO_2_ conditions to compare the labelling kinetics of ADPG with UDPG. The latter nucleotide-sugar is the substrate for sucrose biosynthesis and potential product of sucrose cleavage by SUS in the cytosol. ADPG was labelled much more rapidly and more completely than UDPG, with very similar labelling kinetics to Calvin-Benson cycle intermediates (Supplementary Fig. 1 and Table 1).

### Generation and analysis of sextuple *sus123456* mutants

Based on localization of the SUS1–SUS4 isoforms^48,49^, the quadruple *sus1234* mutant is expected to lack SUS activity in the mesophyll cells where transitory starch is synthesized^57^. The residual SUS activity in this mutant is disputed, with Barratt et al.^48^ reporting that soluble SUS activities in roots and stems of the quadruple *sus1234* mutant were less than 2% of those in WT plants, whereas Baroja-Fernández et al.^25^ reported soluble SUS activities of 98% and 90% of WT in leaves and stems of the *sus1234* mutant. To resolve these discrepancies, we generated two independent sextuple *sus1 sus2 sus3 sus4 sus5 sus6* (*sus12345*^*1*^*6* and *sus12345*^*2*^*6*) mutants by CRISPR/Cas9-mediated gene editing of the *SUS5* and *SUS6* genes in the *sus1234* mutant background. The CRISPR (clustered regularly interspaced short palindromic repeats)/Cas9 (CRISPR-associated protein 9)-generated mutations in the *SUS5* (*sus5-1* and *sus5-2*) and *SUS6* (*sus6*) genes gave rise to premature stop codons (Supplementary Fig. 2) within the respective regions coding for the catalytic glucosyltransferase domains of the enzymes. The zygosity of the *sus5* and *sus6* alleles was tested by genomic PCR using gene-specific primers followed by restriction enzyme digestion of the PCR products (Supplementary Fig. 3). The presence of homozygous T-DNA insertions in the *SUS1, SUS2, SUS3* and *SUS4* genes was confirmed in both of the gene-edited lines (Supplementary Fig. 3), establishing that the homozygous sextuple mutants (*sus12345*^*1*^*6* and *sus12345*^*2*^*6*) lacked functional copies of all six *Arabidopsis* *SUS* genes.

**Fig. 4:**
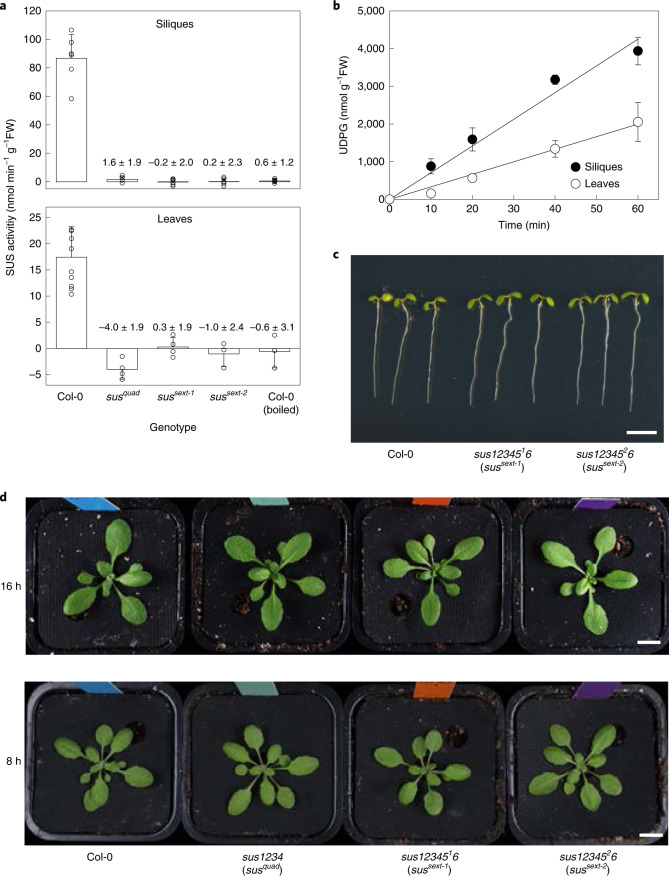
Sucrose synthase activity and morphology of wild-type and *sus* mutant *Arabidopsis*. **a**, Sucrose synthase activity of developing siliques and rosettes, measured as the UDP-dependent production of UDP-glucose from sucrose. Bars show mean ± s.d. from 6 independently pooled siliques (*n* = 6 biological replicates) or independently pooled batches of 5 rosettes from Col-0 (*n* = 10 biological replicates), *sus*^*quad*^(*n* = 4 biological replicates), *sus*^*sext-1*^ (*n* = 4 biological replicates), *sus*^*sext-2*^ (*n* = 3 biological replicates) and Col-0 boiled (*n* = 3 biological replicates). **b**, Linearity of sucrose synthase assay. Datapoints are shown as mean ± s.d. of 3 technical replicates from single wild-type Col-0 silique and rosette extracts. **c**, Seedling morphology at 5 d after germination on 0.5× MS medium. Scale bar, 5 mm. (**d**) Rosette morphology of plants grown in a 16 h photoperiod for 3 weeks or an 8 h photoperiod for 4 weeks. Scale bars, 1 cm. *sus*^*quad*^, *sus1234*; *sus*^*sext-1*^, *sus12345*^*1*^*6*; *sus*^*sext-2*^, *sus12345*^*2*^*6*.

To confirm loss of SUS activity, we measured activity in developing siliques and in rosette leaves of WT, *sus1234* and the two sextuple *sus123456* mutants. Developing siliques were chosen initially because all six *SUS* genes are expressed in siliques and/or seeds, as visualized using the Plant eFP browser (https://bar.utoronto.ca/eplant; Supplementary Fig. 4)^58,59^, and SUS activity is high and readily quantified in these tissues^60^. Activity was determined as the UDP-dependent production of UDP-glucose from sucrose, with UDP-glucose being measured enzymatically as previously described^61^. The soluble SUS activity in WT Col-0 siliques was 86.8 ± 16.7 nmol min^−1^ g^−1^ FW (Fig. 4a) and the assay was linear with time for at least 40 min (Fig. 4b). Activities were much lower or undetectable in the *sus1234* (1.6 ± 1.9 nmol min^−1^ g^−1^ FW; <2% of WT), *sus12345*^*1*^*6* (−0.2 ± 2.9 nmol min^−1^ g^−1^ FW; that is, no detectable activity) and *sus12345*^*2*^*6* (0.2 ± 2.0 nmol min^−1^ g^−1^ FW; <0.2% of WT) mutants (Fig. 4a). The soluble SUS activity in WT Col-0 rosettes was 17.4 ± 5.9 nmol min^−1^ g^−1^ FW (Fig. 4a) and the assay was linear with time for at least 60 min (Fig. 4b). Activities were much lower or undetectable in the *sus1234* (−4.0 ± 1.9 nmol min^−1^ g^−1^ FW; that is, no detectable activity), *sus12345*^*1*^*6* (0.3 ± 1.9 nmol min^−1^ g^−1^ FW; <2% of WT) and *sus12345*^*2*^*6* (−1.0 ± 2.4 nmol min^−1^ g^−1^ FW; that is, no detectable activity) mutants (Fig. 4a). The residual activities detected in extracts of *sus12345*^*2*^*6* siliques and *sus12345*^*1*^*6* rosettes were within the range of values obtained with a boiled extract of Col-0 siliques (0.6 ± 1.2 nmol min^−1^ g^−1^ FW; Fig. 4), indicating that the apparent activities simply reflect noise at the detection limit of the assay. We conclude that the sextuple *sus123456* mutants have no detectable soluble SUS activity. The *sus123456* mutants showed WT-like growth under standard laboratory conditions (Fig. 4c,d), indicating that SUS is not essential for plant survival under benign growth conditions.

**Fig. 5:**
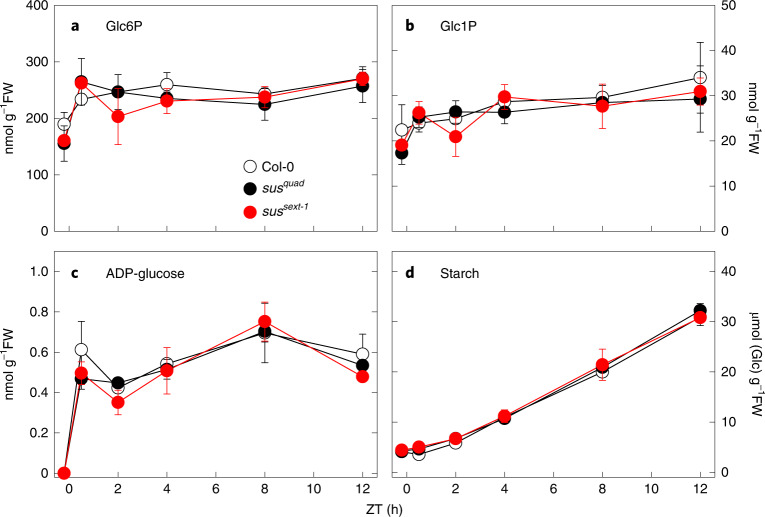
Metabolite levels in wild-type and *sus* mutant *Arabidopsis*. **a**–**d**, Wild-type Col-0, *sus1234* mutant (*sus*^*quad*^) and *sus12345*^*1*^*6* (*sus*^*sext-1*^) mutants were grown in long-day conditions (16 h photoperiod). At 25 d after germination, rosettes were harvested just before dawn (ZT0.2) and at intervals from ZT0.5 to ZT12 for measurement of (**a**) glucose 6-phosphate (Glc6P), (**b**) glucose 1-phosphate (Glc1P), (**c**) ADP-glucose (ADPG) and (**d**) starch. Data are mean ± s.d. from 3 independently pooled batches of 5 rosettes (*n* = 3 biological replicates). *P* values for all genotype × genotype comparisons are shown in Supplementary Table 3.

WT Col-0, *sus1234* and *sus12345*^*1*^*6* plants were grown under long-day (16 h photoperiod) conditions and rosettes were harvested just before dawn and at intervals during the day for metabolite analysis. In WT Col-0 plants, Glc6P, Glc1P and ADPG increased upon illumination (Fig. 5a–c), as observed previously (Fig. 3), and after a slight lag the plants accumulated starch in a linear manner (Fig. 5d). The levels and diel patterns of Glc1P, Glc6P, ADPG and starch were essentially identical in the *sus1234* and *sus12345*^*1*^*6* plants and not significantly different from those of WT plants (Fig. 5 and Supplementary Table 2). Likewise, there were no significant differences between the mutant and WT Col-0 plants in the levels of soluble sugars (sucrose, glucose and fructose) or intermediates of sucrose synthesis (Fru6P, UDP-glucose, dihydroxyacetone-phosphate; Supplementary Fig. 5 and Table 3). In an independent experiment under the same growth conditions, *sus12345*^*2*^*6* plants had levels and diel patterns of starch and soluble sugars that were essentially identical to those in WT plants (Supplementary Fig. 6).

## Discussion

According to the consensus pathway, the starch-deficient *pgm* and *adg1* mutants should have little or no ADPG due to loss of essential enzymes for its synthesis in the chloroplasts. Reports of near-WT levels of ADPG in these two mutants^28,29^ appeared to be inconsistent with expectations. To reassess these reports, we established a robust method for harvesting *Arabidopsis* rosettes in the light to maintain in vivo levels of metabolites such as ADPG that are present in very small amounts and turn over very quickly (half time <1 s)^56^. We used LC–MS/MS^55,56^ to assay ADPG with greater sensitivity and specificity than the HPLC-UV/HPLC-PAD methods that were used for most previous studies. We found that shading of the leaves of WT plants for just a few seconds leads to a rapid decrease in measurable ADPG content, down to levels that resemble those found in the starch-deficient *pgm* and *adg1* mutants. Consequently, unless extreme care is taken during sample collection, ADPG levels are likely to be underestimated and therefore unrepresentative of the levels during steady-state photosynthesis. This issue may well have affected values presented in earlier papers, including some of our own.

Using our optimized methods, we found that ADPG levels in illuminated leaves of the *pgm* and *adg1* mutants were <6% and <4% of WT, respectively, but the same as WT in the *sus1234* mutant (Fig. 3c). In contrast, and using an LC–MS-based method similar to our own, Bahaji et al.^29^ reported that ADPG levels were the same in WT leaves and leaves of the *pgm* and *aps1* mutants (*aps1*, similar to *adg1*, lacks a subunit of AGPase and is nearly starchless). In earlier work that employed the HPLC-UV assay for ADPG, these researchers reported that *adg1* leaves also had ADPG levels comparable with those of WT leaves^28^. One possible explanation for this discrepancy between our results and those of Bahaji and colleagues is that their plants were inadvertently shaded during harvest, potentially leading to large losses of ADPG before quenching. Consistent with this idea is the fact that their reported values for ADPG in WT leaves (0.13 nmol g^−1^ FW)^29^ are substantially lower than the range we observed in WT leaves (0.5–3 nmol g^−1^ FW; Figs. 2a, 3c and 5c). Their low value for WT leaves is comparable with the amounts we observed after 2–3 s of shading, and with our shaded values for *pgm* and *adg1*. Thus, we suspect that the similar ADPG values for WT and mutant plants reported by Bahaji et al.^29^, and the discrepancies between their results and ours are due to loss of ADPG during harvesting of WT leaves in their experiments.

Our data are entirely consistent with predictions based on the consensus pathway being the predominant, if not the only, pathway of starch synthesis in leaves. Given the precautions we took to ensure that in vivo metabolite levels were preserved during tissue sampling and the robustness of the LC–MS/MS assay for ADPG, we would argue that our results are more trustworthy than those used by others to question the validity of the consensus pathway.

The soluble SUS activity in WT Col-0 rosettes, measured under linear conditions with an established assay (Fig. 4b), was 17.4 ± 5.9 nmol min^−1^ g^−1^ FW (Fig. 4a), which is too low to account for the observed rates of starch synthesis in WT leaves under our experimental conditions (approx. 40 nmol [Glc] min^−1^ g^−1^ FW; Figs. 3d and 5d,e). Nevertheless, this does not exclude the possibility of a minor contribution to starch synthesis from ADPG produced via SUS. Therefore, to test whether the proposed alternative pathway of ADPG synthesis via SUS makes any significant contribution to starch synthesis, we re-analysed the *sus1234* mutant reported in Barratt et al.^48^ and also generated two sextuple *sus123456* mutants that lack all known isoforms of SUS in *Arabidopsis*.

The *sus1234* mutant lacks *SUS* expression in mesophyll cells where transitory starch is made in WT plants^48,57^. The mutant has little or no detectable soluble SUS activity in developing siliques, where activity is high and readily measurable in WT plants, or in rosette leaves (Fig. 4a). The *sus1234* mutant should have less starch than WT plants if the SUS pathway supplies a significant amount of ADPG for starch synthesis. However, we confirmed that starch levels in this mutant are indistinguishable from that of WT, as reported in Barratt et al.^48^ and Baroja-Fernández et al.^25^, and showed that the mutant also has WT levels of ADPG and of the two other intermediates of the consensus pathway, Glc6P and Glc1P (Figs. 3 and 5). Likewise, sextuple *sus123456* mutants that had no detectable SUS activity in developing siliques or rosettes contained WT levels of starch (Fig. 4e and Supplementary Fig. 5a). Key intermediates from the consensus pathway for starch synthesis (Glc6P, Glc1P, ADPG) were also essentially the same as in WT (Fig. 4a–c), as were soluble sugars and intermediates of sucrose synthesis (dihydroxyacetone phosphate, Fru6P, UDPG) (Fig. 5, and Supplementary Figs. 5 and 6).

In principle, it is conceivable that SUS does contribute to ADPG formation and starch synthesis in wild-type leaves, but that in the quadruple and sextuple *sus* mutants this function can be completely substituted by the consensus pathway via chloroplastic ADPG pyrophosphorylase. However, two observations make this extremely improbable. First, it has previously been shown that essentially all of the ADPG in *Arabidopsis* leaves is located in the chloroplasts^53^. Second, pulse-labelling with ^13^CO_2_ showed that ADPG is rapidly labelled with similar kinetics to Calvin-Benson cycle intermediates (Supplementary Fig. 1), confirming previous observations^53^. Labelling of ADPG was both more rapid and more complete than labelling of UDPG, the precursor of sucrose and potential product of sucrose cleavage via SUS (Supplementary Fig. 1). Together, these results show that SUS is not required for transitory starch synthesis in *Arabidopsis* leaves, and argue strongly against the proposed SUS pathway making any significant contribution to ADPG and starch synthesis in WT leaves.

## Methods

### Plant materials

*Arabidopsis* (*Arabidopsis thaliana* [L.] Heynh) Columbia-0 wild-type and the *pgm*^5^, *adg1*^7^ and *sus1234*^48^ mutant germplasm were from in-house collections.

Sextuple *sus123456* mutants were generated by gene editing of the *SUS5* and *SUS6* genes in the *sus1234*^48^ background using CRISPR/Cas9 and two pairs of guide RNA (gRNA) targets: *SUS5*—GAAATGACATCTGGATCGTT and TGTAGAACTTGGTGAATCTC; *SUS6—*GGTAAGGGTAGATATCGAAT and

GTTCTTGAAGCACCAGACAA. The CRISPR/Cas9 and gRNA sequences were cloned into the pHEE401E vector as previouslly described^62,63^. The construct was introduced into the *sus1234* quadruple mutant by *Agrobacterium*-mediated transformation using the floral-dip method^64^ to generate sextuple *sus123456* mutants. The CRISPR/Cas9 construct was subsequently eliminated by crossing the sextuple *sus123456* lines with the quadruple *sus1234* parent. Progeny were screened by PCR to identify lines that were homozygous for the T-DNA insertions in *SUS1–SUS4* and for gene-edited mutant alleles of *SUS5* and *SUS6* (Supplementary Fig. 3). Mutations in the *SUS5* gene were identified by PCR using primers sus5-Fw and sus5-Rv (Supplementary Table 4) and restriction with HinfI. Mutations in the *SUS6* gene were identified by PCR using primers sus6-Fw and sus6-Rv (Supplementary Table 4) and restriction with Van91I (Supplementary Fig. 3).

Plants were grown in a 1:1 mixture of compost and vermiculite in 6-cm-diameter pots with a 16 h photoperiod (140 μE m^−2^ s^−1^ irradiance provided by white fluorescent tubes) and day/night temperatures of 20 °C/18 °C. For the ^13^CO_2_-labelling experiment, plants were grown with a 12 h photoperiod (120 µE m^−2^ s^−1^ irradiance). Seedlings were also grown on agar plates containing 0.5× Murashige–Skoog medium.

### Enzyme activity measurements

Finely ground frozen leaf tissue (20 mg) was homogenized in 1 ml of ice-cold extraction buffer as previously described^65^. After centrifugation of the crude extract at 10,000 × *g* (4 °C) for 5 min, an aliquot (180 µl) of the supernatant was desalted by centrifugation through a MicroSpin column (GE Healthcare Life sciences) equilibrated with extraction buffer. Soluble SUS activity was determined in the sucrose cleavage direction by measuring the UDP-dependent production of UDPG from sucrose, with enzymatic determination of UDPG as previously described^61^. Reaction mixtures (225 µl) contained: 20 mM PIPES-KOH (pH 6.5), 3 mM MgCl_2_, 100 mM sucrose and 2 mM UDP, with UDP being omitted from blank reactions. Reactions were started by addition of 25 µl of desalted extract and incubation at 30 °C for 25 min. The reaction was stopped by addition of 250 µl 50 mM Tricine-KOH (pH 8.3) and heating at 100 °C for 2 min. UDPG was measured enzymatically by coupling to reduction of NADP^+^ and monitoring the increase in absorbance at 340 nm. The initial reaction mixture (500 µl) contained: 50 mM Tricine-HCl (pH 8.0), 4 mM MgCl_2_, 12.5 mM NADP+, 0.3 U glucose-6-phosphate dehydrogenase (EC 1.1.1.49; from yeast), 0.4 U phosphoglucomutase (EC 5.4.2.2; from rabbit muscle) and 400 µl of the SUS assay reaction. When the A340 was stable, UDPG was determined by addition of sodium pyrophosphate (final concentration 1 mM) and 0.2 U UDP-glucose pyrophosphorylase (EC 2.7.7.9; from yeast). All coupling enzymes were from Sigma-Aldrich.

### Metabolite measurements

Rosettes were rapidly quenched in the light by either pouring liquid nitrogen directly onto the rosettes, or by cutting the hypocotyl from below and rapidly transferring the cut rosette into liquid nitrogen in the light, taking care not to shade the leaves at any time. Phosphorylated intermediates were extracted using chloroform-methanol as previously described^55^ and measured by ion-pair reverse phase LC–MS/MS^56^ or anion-exchange LC–MS/MS^55^ (with modifications as previously described^66^). Soluble sugars were measured in ethanolic extracts and starch was measured in the ethanol-insoluble residue as previously described^67^.

### ^13^CO_2_ pulse labelling

Four-week-old WT *Arabidopsis* Col-0 plants were pulse-labelled with ^13^CO_2_ (400 µl l^−1^) at an irradiance of 120 µE m^−2^ s^−1^, for 0, 1 or 20 min, and rapidly quenched with liquid nitrogen under ambient illumination as previously described^53^. Labelling was performed between ZT4 and ZT7. Isotopomers of nucleotide sugars and phosphorylated intermediates were quantified by LC–MS/MS^53^.

### Statistical analysis

Statistical analysis was performed using Microsoft Excel 2019 MSO (https://www.microsoft.com) or Sigmaplot for Windows version 14.5 (Systat Software; http://www.systat.de).

### Reporting Summary

Further information on research design is available in the Nature Research Reporting Summary linked to this article.

## Supplementary information

Supplementary Information Supplementary Figs. 1–6 and Tables 2–4.

Reporting Summary.

Supplementary Table 1 ^13^C-labelling data from wild-type *Arabidopsis* Col-0 leaves.

## Data Availability

Original data and *Arabidopsis* lines are available upon request from the corresponding authors.
