# Supplementary material for: Sucrose synthases are not involved in starch synthesis in Arabidopsis leaves
Source: Nat Plants. 2022 Apr 28;8(5):574–82. doi: 10.1038/s41477-022-01140-y (PMC9122829; doi:10.1038/s41477-022-01140-y)
Supplement: Supplementary file 1 — Supplementary Figs. 1–6 and Tables 2–4. [file 41477_2022_1140_MOESM1_ESM.pdf]

nature portfolio

https://doi.org/10.1038/s41477-022-01140-y

Supplementary information

Sucrose synthases are not involved in starch synthesis in *Arabidopsis* leaves

In the format provided by the authors and unedited

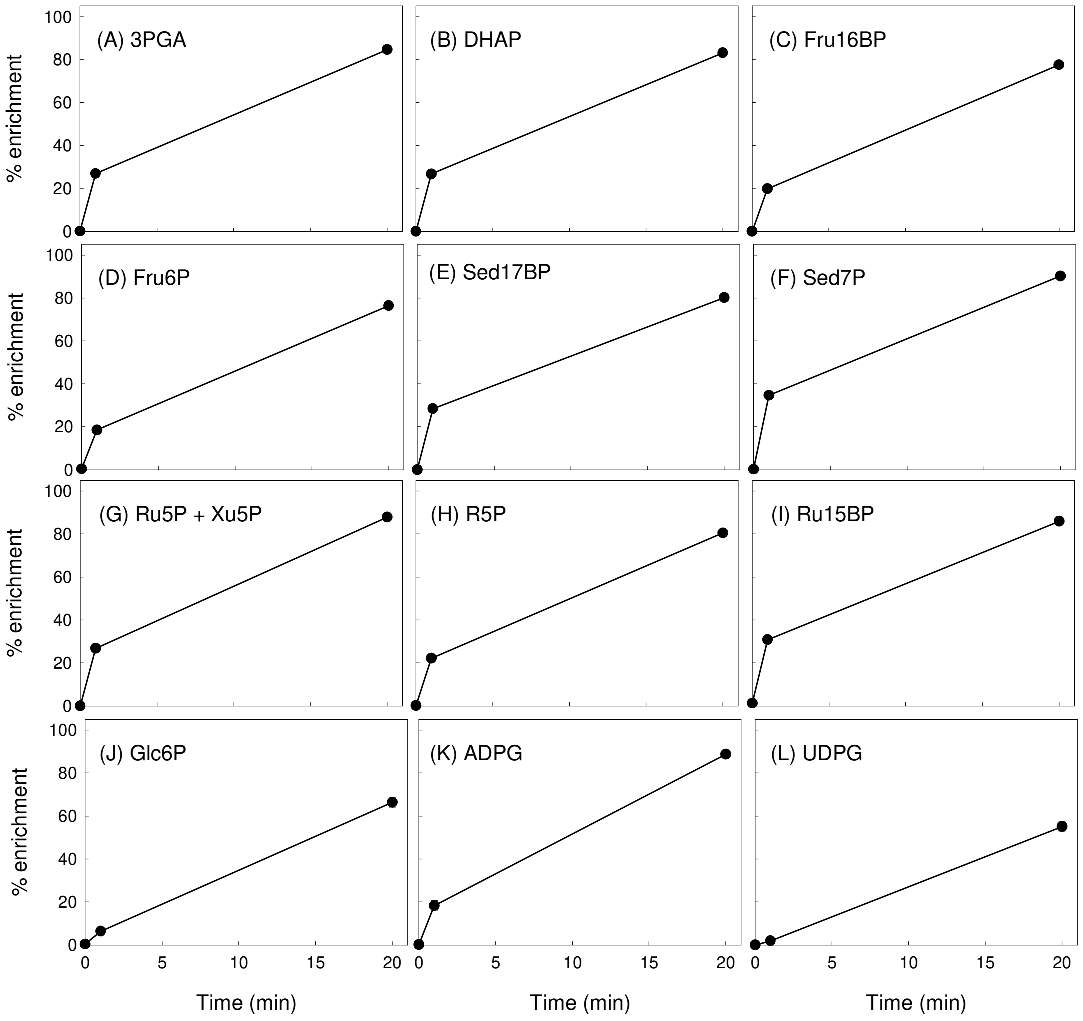

Supplementary Figure S1. ^{13}C-labelling kinetics of phosphorylated intermediates and nucleotide sugars in wild-type Arabidopsis Col-0 leaves

Plants were grown in 12-h photoperiod. At 28 days after germination, rosettes were pulse labelled with $^{13}CO_2$ (400 ppm) in the light (irradiance 150 µmol m^{-2} s^{-1}) for 0, 1 or 20 min. (between ZT4-ZT7) and isotopomers were quantified by LC-MS/MS as described in Szecowka et al. (2013) to calculate % enrichment (glucose moiety only for nucleotide sugars). Data are mean ± S.D. from three individual rosettes (n=3 biological replicates). Abbreviations: 3PGA, 3-phosphoglycerate; DHAP, dihydroxyacetone-phosphate; Fru16BP, fructose-1,6-bisphosphate; Fru6P, fructose 6-phosphate; Sed17BP, sedoheptulose-1,7-bisphosphate, Sed7P, sedoheptulose 7-phosphate; Ru5P + Xu5P, ribulose 5-phosphate + xylulose 5-phosphate; R5P, ribose 5-phosphate; Ru15BP, ribulose-1,5-bisphosphate; Glc6P, glucose 6-phosphate; ADPG, ADP-glucose; UDPG, UDP-glucose. Supplementary Table S1 contains the original data.

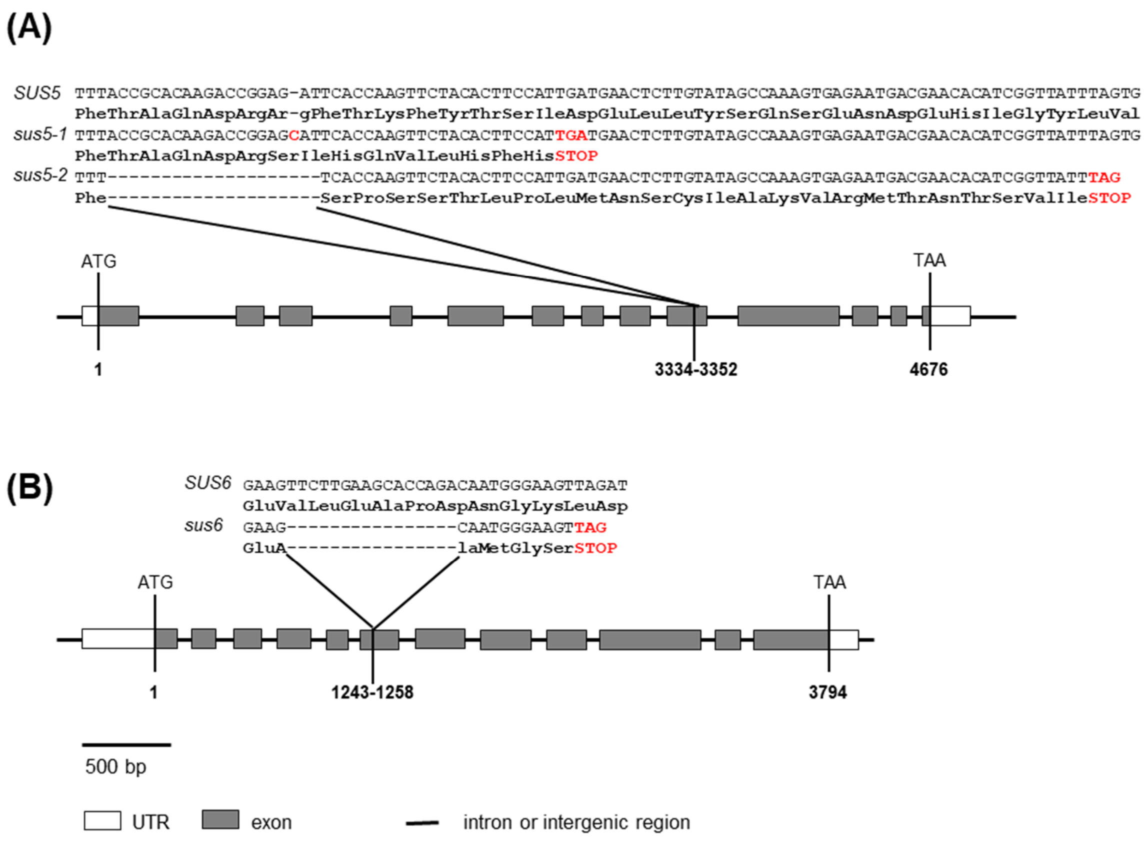

Supplementary Figure S2. Schematic diagram of the gene edited mutations in the Arabidopsis *SUS5* and *SUS6* genes.

(A) *SUS5* gene showing the wild-type (Col-0) sequence, the *sus5-1* allele containing a single base (C) insertion (shown in red) and the *sus5-2* allele containing a 20-bp deletion. (B) *SUS6* gene showing the wild-type (Col-0) sequence and the *sus6* allele containing a 16-bp deletion. All mutations generated premature STOP codons (shown in red). The dark grey and white boxes indicate translated and untranslated regions, respectively.

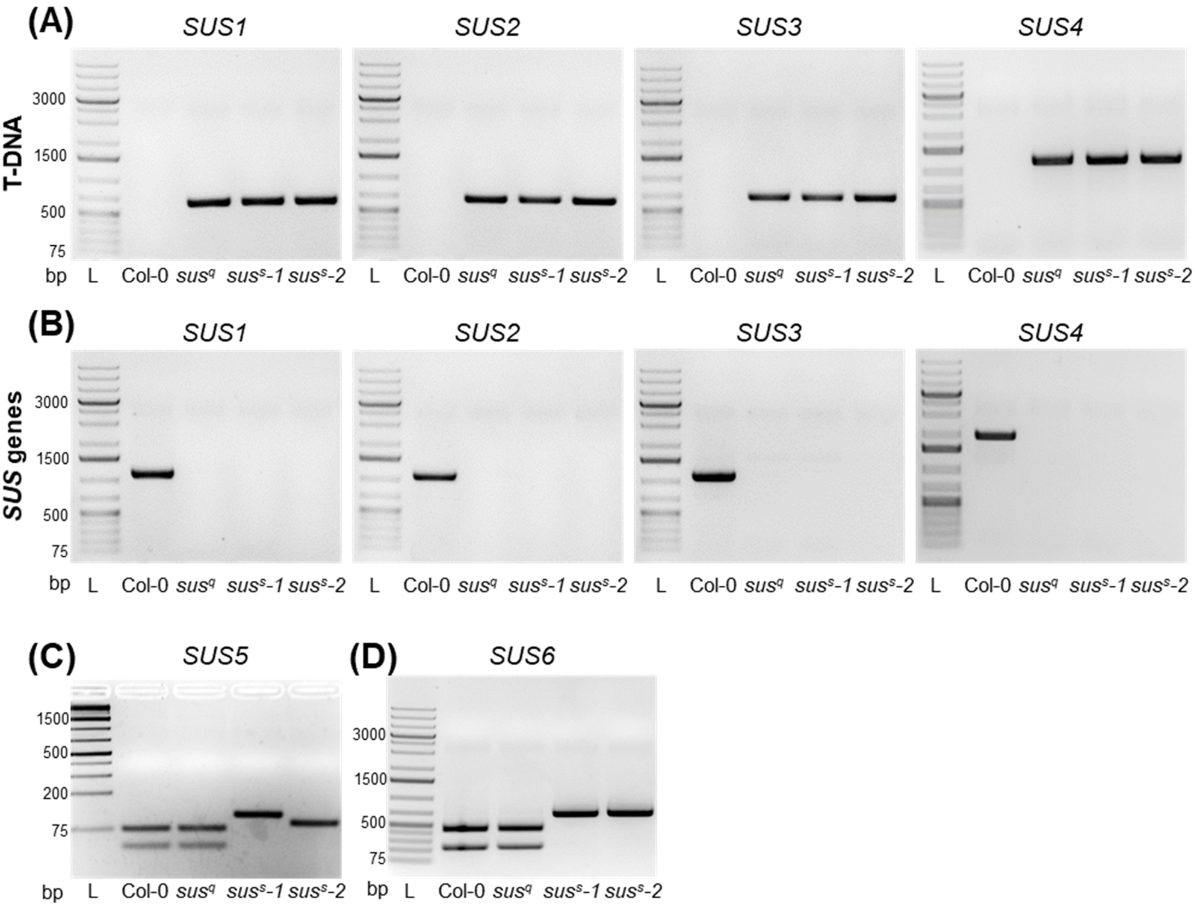

Supplementary Figure S3. PCR genotyping of Arabidopsis *sus* mutants.

(A) T-DNA insertions in the *SUS1*, *SUS2*, *SUS3*and *SUS4* genes were confirmed by PCR using gene specific (LP) and T-DNA border primers (LBb1.3 for *sus1*, *sus2*, *sus3* and P66 for *sus4*). (B) Homozygosity of T-DNA insertion loci in the *SUS1*, *SUS2*, *SUS3* and *SUS4* genes was confirmed by genomic PCR using *SUS* gene specific primers (LP and RP) that flank the respective T-DNA insertions. (C) Identification of mutations in the *SUS5* gene by genomic PCR (using primers sus5-Fw and sus5-Rv) and restriction with HinfI, yielding two fragments (75 and 37 bp) from the wild-type *SUS5* gene. (D) Identification of mutations in the *SUS6* gene by genomic PCR (using primers sus6-Fw and sus6-Rv) and restriction with Van91I, yielding two fragments (471 and 235 bp) from the wild-type *SUS6* gene. Abbreviations: L, GeneRuler 1-kb Plus DNA Ladder (Thermo Scientific); Col-0, wild type Columba-0; Sus^q, *sus1234* quadruple mutant; sus^s*-1*, $sus12345^16$ sextuple mutant; sus^s*-2*, $sus12345^26$ sextuple mutant. PCR genotyping was performed twice for the *SUS1*-*SUS4* genes (A-B) and three times for the *SUS5* (C) and *SUS6* (D) genes with consistent results.

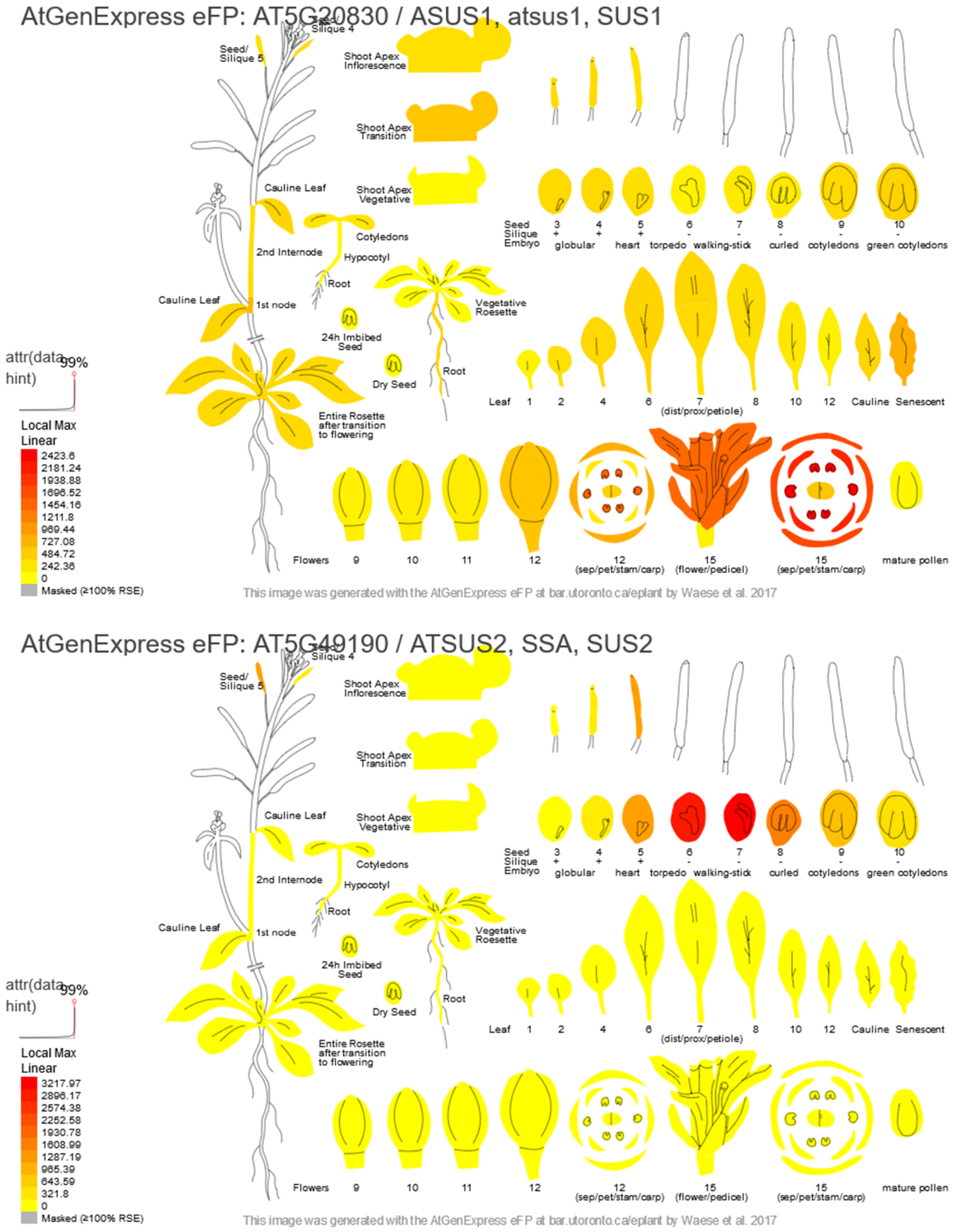

Supplementary Figure S4a. Expression atlas of Arabidopsis *SUS1* and *SUS2* transcripts.

SUS1 (At5g20830) and *SUS2* (At5g49190) transcript abundance in different Arabidopsis tissues and developmental stages based on Affymetrix ATH1 array data from Schmid et al. (2005), and visualized with the Plant eFP browser (https://bar.utoronto.ca/eplant; Winter et al. (2007); Waese et al. (2017).

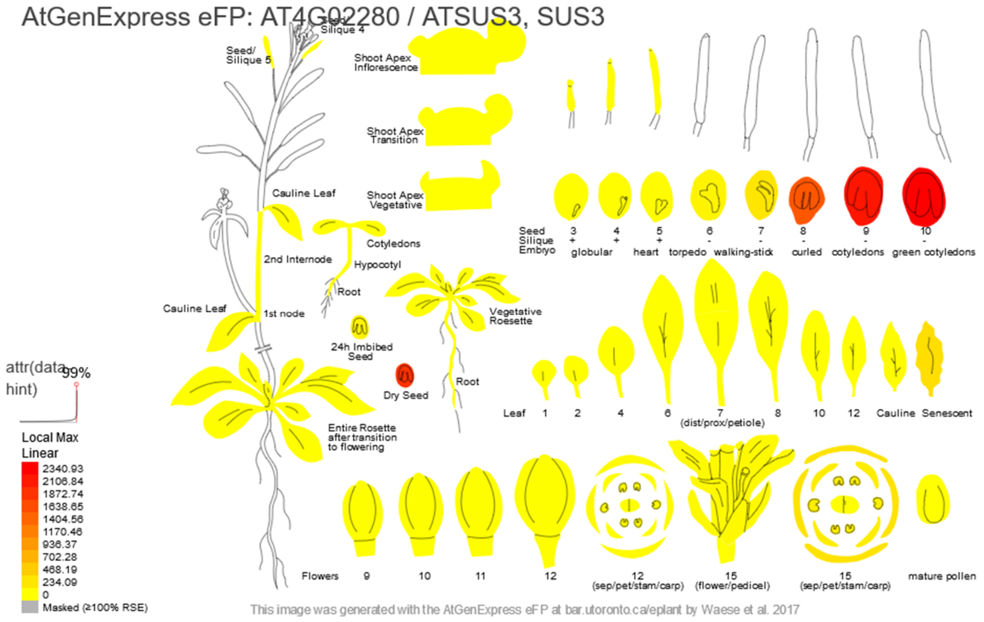

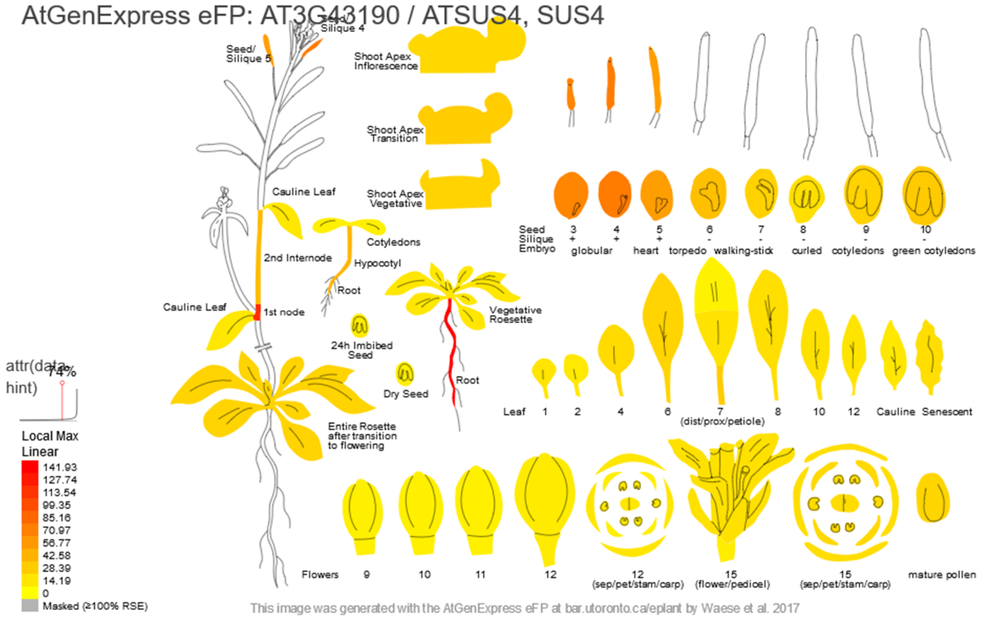

Supplementary Figure S4b. Expression atlas of Arabidopsis *SUS3* and *SUS4* transcripts.

SUS3 (At4g02280) and *SUS4* (At3g43190) transcript abundance in different Arabidopsis tissues and developmental stages based on Affymetrix ATH1 array data from Schmid et al. (2005), and visualized with the Plant eFP browser (https://bar.utoronto.ca/eplant; Winter et al. (2007); Waese et al. (2017).

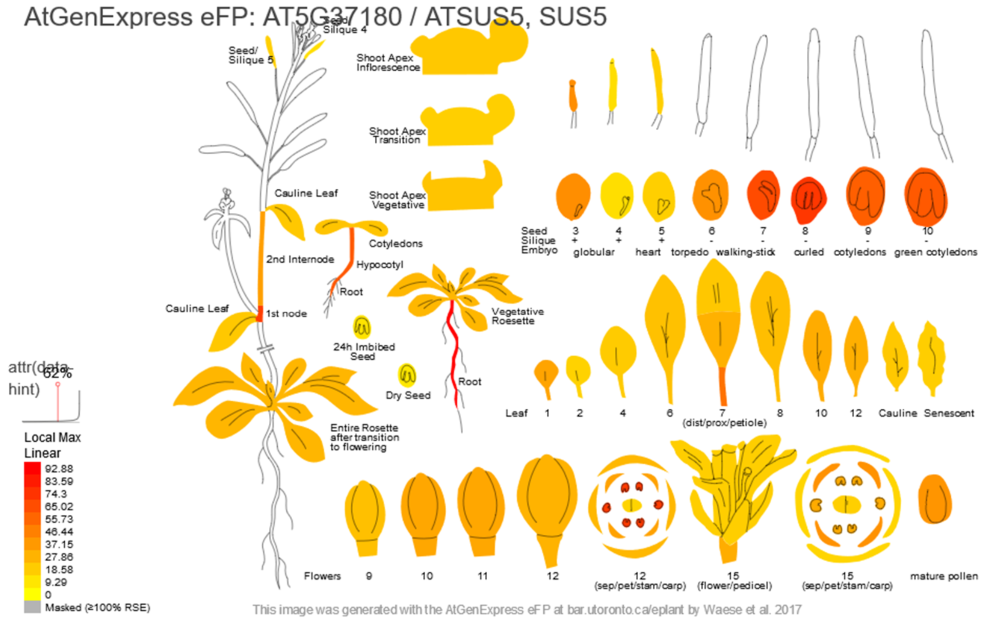

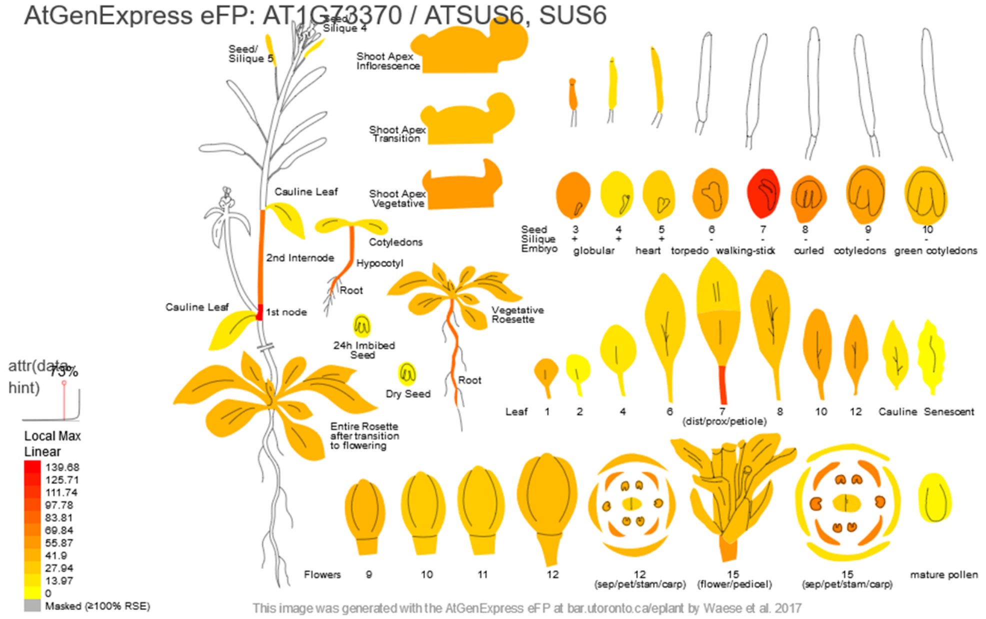

Supplementary Figure S4c. Expression atlas of Arabidopsis *SUS5* and *SUS6* transcripts.

SUS5 (At5g37180) and *SUS6* (At1g73370) transcript abundance in different Arabidopsis tissues and developmental stages based on Affymetrix ATH1 array data from Schmid et al. (2005), and visualized with the Plant eFP browser (https://bar.utoronto.ca/eplant; Winter et al. (2007); Waese et al. (2017).

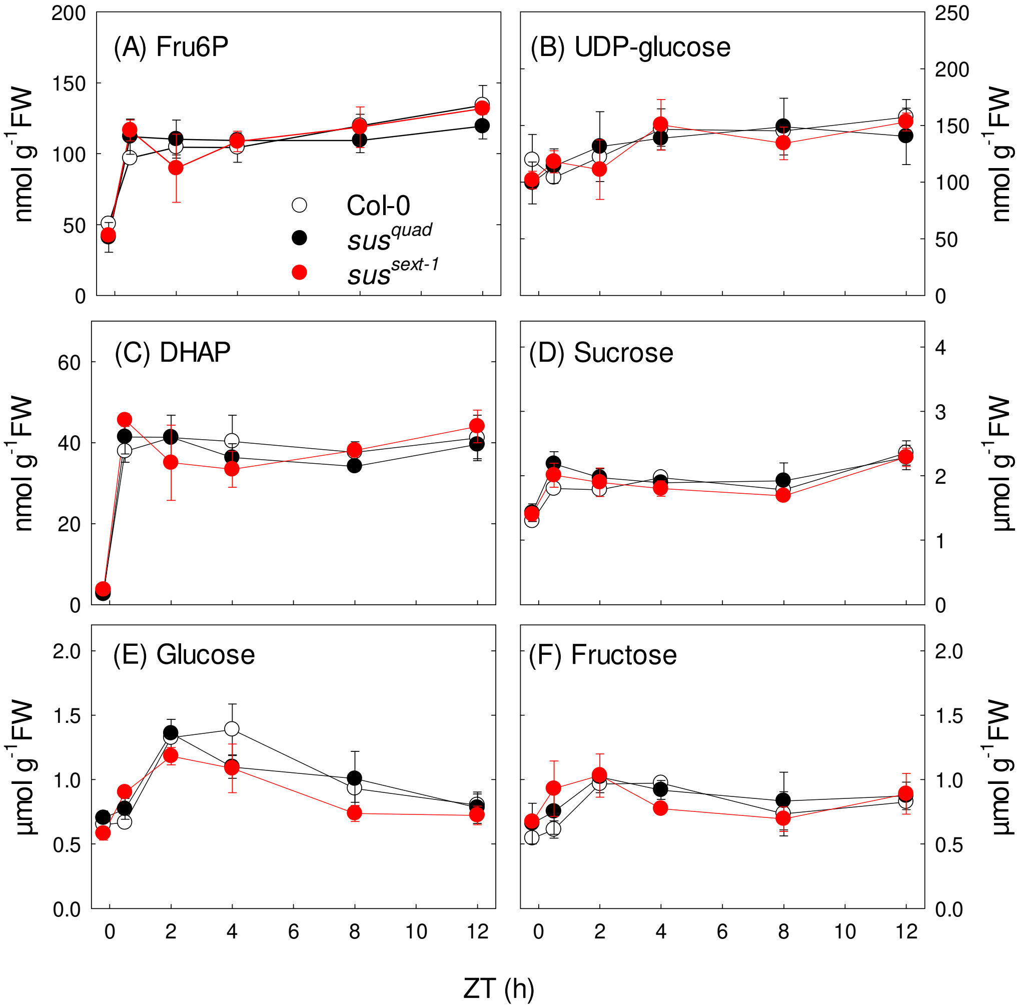

Supplementary Figure S5. Metabolite levels in wild-type Arabidopsis and *sus* mutants.

Wild-type Col-0, and the *sus1234* mutant (*sus*^{quad}) and *sus12345*^{1}*6* (*sus*$^{sext\text{-}1}$) mutants were grown in long-day conditions (16-h photoperiod). At 25 days after germination, rosettes were harvested just before dawn (ZT-0.2) and at intervals from ZT0.5 to ZT12 for measurement of: (A) fructose 6-phosphate (Fru6P), (B) UDP-glucose, (C) dihydroxyacetone-phosphate (DHAP), (D) sucrose, (E) glucose and (F) fructose. Data are from the same experiment as Fig. 5 and presented as mean ± S.D. (*n*=3 biological replicates). *P*-values for all genotype x genotype comparisons are shown in Supplementary Table S3.

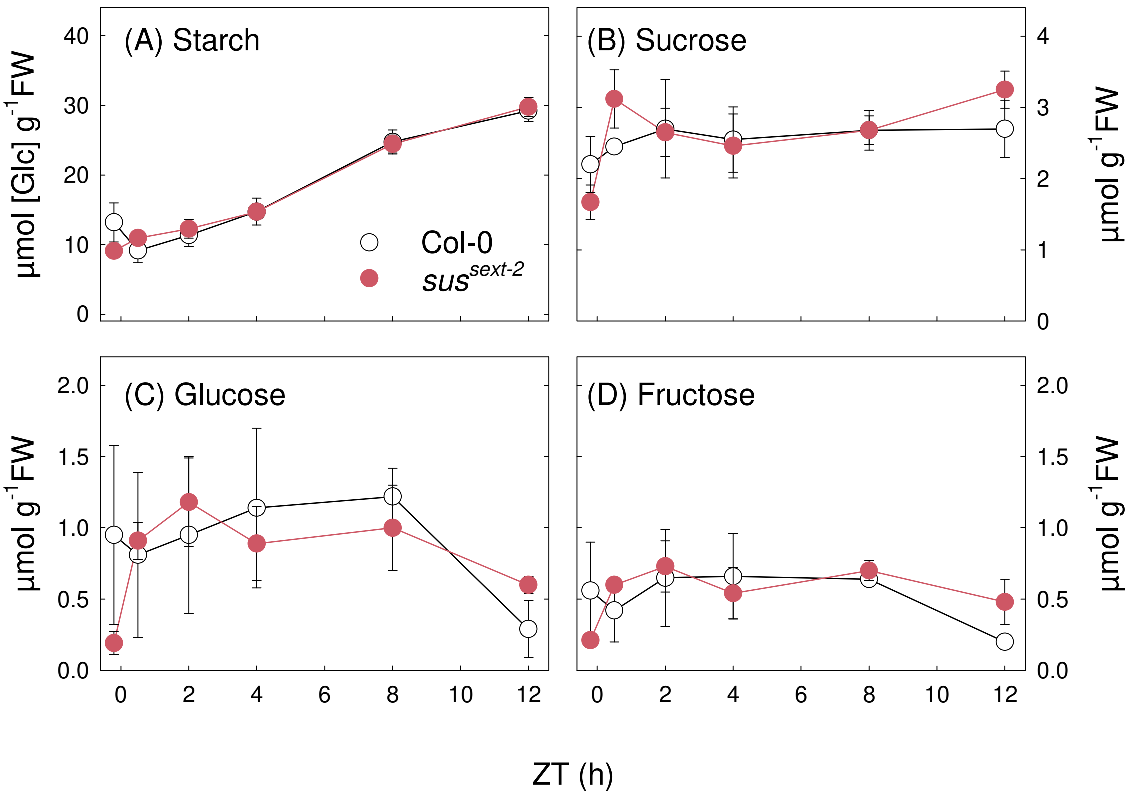

Supplementary Figure S6. Starch and sugar levels in wild-type Arabidopsis (Col-0) and the *sus12345²6* mutant (*sus^{sext-2}*)

Plants were grown in long-day conditions (16-h photoperiod). At 25 days after germination, rosettes were harvested just before dawn (ZT-0.2) and at intervals from ZT0.5 to ZT12 for measurement of: (A) starch, (B) sucrose, (C) glucose and (D) fructose. Data are mean ± S.D. from three independently pooled batches of five rosettes (*n*=3 biological replicates).

Supplementary Table S2. Statistical analysis of metabolite data in Figure 3.

Metabolite levels in rosettes of Col-0, *sus1234* (*sus*^{quad}), *pgm* and *adg1* were compared across all sampling times by two-way ANOVA with Tukey's honestly significant difference test. In columns 2-5, letters indicate significant differences between genotypes, with the *P*-value shown in column 6 and level of significance in column 7. **P*<0.05; ***P*<0.01; ****P*<0.001; n.s., not significant.

**Metabolite**	**Col-0**	***sus*^{quad}**	***pgm***	***adg1***	***P*-value**	**Significance**
Glc6P	b	ab	a	a	<0.001	***
Glc1P	bc	ab	c	a	0.166	n.s.
ADP-Glc	a	a	b	b	<0.001	***
Starch	a	a	b	b	<0.001	***

Supplementary Table S3. Statistical analysis of metabolite data in Figure 5 and Supplemental Figure S5.

Metabolite levels in rosettes of Col-0, *sus1234* (*sus*^{quad}) and *sus123456* (*sus*^{sext}*-1*) were compared across all sampling times by two-way ANOVA with Tukey's honestly significant difference test. In columns 2-4, letters indicate genotypes that were not significantly different for a given metabolite, with the *P*-value shown in column 5 and level of significance in column 6. n.s., not significant.

Metabolite	Col-0	*sus*^{quad}	*sus*^{sext}*-1*	*P*-value	Significance
Glc6P	a	a	a	0.518	n.s.
Glc1P	a	a	a	0.382	n.s.
ADPG	a	a	a	0.774	n.s.
Starch	a	a	a	0.228	n.s.
Fru6P	a	a	a	0.981	n.s.
UDPG	a	a	a	0.752	n.s.
DHAP	a	a	a	0.973	n.s.
Sucrose	a	a	a	0.360	n.s.
Glucose	a	a	a	0.535	n.s.
Fructose	a	a	a	0.471	n.s.

Supplementary Table S4. Oligonucleotide primers for PCR genotyping.

Primer	Sequence (5´→3´)
SUS genomic primers	
SUS1-LP	ATGGCAAACGCTGAACGTATG
SUS1-RP	CTCAAGAGTGCAAGGATCAGG
SUS2-LP	ATGCGGAGACAAAATCACAAC
SUS2-RP	TAAGACTGTGAAAGTTGATGG
SUS3-LP	ATCGATGTGTTTGATCCGAAG
SUS3-RP	TTGGAGACCAGCGTCTGATAC
SUS4-LP	ATGGCAAACGCAGAACGTGTAA
SUS4-RP	CTTAGTCTTCTCCAAAGCATG
sus5-Fw	CGGATGACTCTATCTATTTCCCT
sus5-Rv	GAGACGTACATGTGTTCGTCATTC
sus6-Fw	ATGCTTGCTGTGATTGTTGTCTCG
sus6-Rv	CGAGGTAAGGGTAGATATCGAATC
T-DNA left border primers	
LBb1.3	ATTTTGCCGATTTCGGAAC
P66	CCCCTGCGCTGACAGCCGGAACACG
